# Improved filtering of DNA methylation microarray data by detection *p* values and its impact on downstream analyses

**DOI:** 10.1186/s13148-019-0615-3

**Published:** 2019-01-24

**Authors:** Jonathan A. Heiss, Allan C. Just

**Affiliations:** Department of Environmental Medicine and Public Health, Icahn School of Medicine at Mount Sinai, One Gustave L. Levy Place, Box 1057, New York, NY 10029 USA

**Keywords:** DNA methylation, Microarray analysis, Illumina 450K, Outlier detection, Data cleaning, EWAS

## Abstract

**Background:**

DNA methylation microarrays are popular for epigenome-wide association studies (EWAS), but spurious values complicate downstream analysis and threaten replication. Conventional cut-offs for detection *p* values for filtering out undetected probes were demonstrated in a single previous study as insufficient leading to many apparent methylation calls in samples from females in probes targeting the Y-chromosome. We present an alternative approach to calculate more accurate detection *p* values utilizing non-specific background fluorescence. We evaluate and compare our proposed approach of filtering observations with conventional ones by assessing the detection of Y-chromosome probes among males and females in 2755 samples from 17 studies on the 450K microarray and masking of large outliers between technical replicates and their impact downstream via an EWAS reanalysis.

**Results:**

In contrast to conventional approaches, ours marks most Y-chromosome probes in females as undetected while removing a median of only 0.14% of the data per sample, catches more (30% vs. 6%) of large outliers (more than 20 percentage point difference between technical replicates), and helps to identify strong associations previously obfuscated by outliers between whole blood DNA methylation and chronological age in a well-powered EWAS (*n* = 729).

**Conclusions:**

We provide guidance for filtering both 450K and EPIC microarrays as an essential preprocessing step to reduce spurious values. An implementation (including a function compatible with objects from the popular *minfi* package) was added to *ewastools*, an R package for comprehensive quality control of DNA methylation microarrays. Scripts to reproduce all analyses are available at doi.org/10.5281/zenodo.1443561.

## Introduction

The Illumina Infinium HumanMethylation450 (450K) and the more recent Infinium MethylationEPIC (EPIC) arrays are two widely popular platforms for epigenome-wide association studies (EWAS). Before beginning with downstream analyses, comprehensive quality control (QC) should be conducted to identify problematic samples. But samples passing QC still contain individual probes with spurious values that do not represent the underlying methylation state, for example, when targeted loci are present in low quantities due to amplification artifacts or are mutated and no longer match their intended complementary probe sequence. Affected probes feature mostly background noise and should be excluded. This decision is based on detection *p* values intended to distinguish signal from noise with a single cut-off. Suggested *p* value cut-offs in the literature span several magnitudes from 0.05 to 1e−16 (the smallest possible floating point double precision number on the arithmetic scale), although 0.05 and 0.01 are most commonly employed. Lehne et al. systematically evaluated detection *p* value cut-offs in a single large study based on the idea that probes targeting the Y-chromosome should be detected in samples from males but not females [1]. Because of an implementation error in the software used by Lehne et al., we reexamined the choice of cut-off by applying their benchmark to a large set of publicly available DNA methylation microarray data from multiple studies and also examine outliers in technical replicates as well as the impact on the findings of a well-powered EWAS. In addition to comparing a wide range of detection *p* value cut-off choices, we demonstrate a modification to computing detection *p* values based on fluorescence resulting from non-specific binding that reflects the background noise distribution more accurately and thus further improves the sensitivity to aberrant values. We provide updated software and guidance for researchers conducting epigenome-wide association studies to easily incorporate this important preprocessing step in their analytical pipeline.

## Methods

Treating DNA with bisulfite converts epigenetic modifications into distinct base sequences. In combination with subsequent whole-genome amplification, the ratio of methylated/unmethylated CpG sites is translated into differences in abundance of these distinct sequences. These hybridize to complementary probes on the 450K and EPIC microarrays and are subsequently linked with fluorescent dyes. By comparing fluorescence intensities of the probes targeting the unmethylated (*U*) and methylated (*M*) variant of a CpG site, its methylation level in the DNA input can be inferred. The Infinium Type I probe design includes separate beads for *U* and *M*, whereas the Infinium Type II design combines *U* and *M* on the same beads but measured with different dyes/color channels [2]. In the case of a completely unmethylated CpG site, the *U* probe features a very high intensity, whereas the *M* intensity is low, or vice versa, in the case of a completely methylated CpG site. Thus, the total fluorescence intensity *T* (the sum of *U* and *M*) for a given site is—to a certain degree—independent of the methylation level itself. High intensities usually indicate good signal-to-noise ratio and such probes are hence deemed *detected*, whereas low intensities consist mostly of background noise and thus are better classified as *undetected* probes. Where to draw the line is based on the concept of detection *p* values: the parameters of a normal background distribution *B* are estimated based on a set of probes thought to feature mainly background noise and a *p* value is computed using a *z* test. If the observed value of *T* is unlikely to be generated by *B* (i.e., when the associated *p* value is below the chosen significance level), a probe is considered detected, otherwise undetected. The previously recommended cut-off of 1e−16 by Lehne et al. is based on *minfi* v1.2.0 [3] which misspecified the parameters of *B* (adding standard deviations instead of variances of the *U* and *M* components of *T*).

We explored two approaches to estimate *B* based on different subsets of probes on the 450K and EPIC microarrays, using either Illumina’s negative control probes specifically designed not to match the human genome, or the fluorescence resulting from non-specific binding observed at the *U* probes for completely methylated and *M* probes for completely unmethylated CpG sites, respectively. Completely (un)methylated CpG sites were identified by selecting the 1000 CpG sites with *β* values closest to each of the two peaks of the bimodal *β* value distribution (locations of peaks were determined as done for *peak-based correction* [4], a within-array normalization method). We picked probes with *β* values close to the peaks instead of from the extremes of the distribution, as the latter would exhibit atypically low intensities of background signal. This was done for both color channels separately and including only probes of Infinium Type I design, as these are only used in a single color channel and thus do not suffer from fluorescence leaking from the other color channel measured at the same bead, i.e., so-called cross talk due to spectral overlap between the two fluorophores used on the 450K/EPIC chip. The markedly compressed dynamic range of Infinium Type II probes [4] is a result of this cross talk because the design relies upon the dye-linked targets binding to the same beads. Indeed, as evidence of cross talk where we would expect no difference in signal, we found across all datasets that for completely unmethylated Type I Red probes the green out-of-band intensities at the *U* beads were a median of 2.7 times higher than those at the *M* beads, indicating that the high red fluorescence at the *U* beads is bleeding into the green color channel. Conversely, for completely methylated Type I Red probes, the green out-of-band intensities at the *M* beads were a median of 3.8 times higher than those at the *U* beads. Robust estimators of location and spread were used with a corrected estimation of the variance of *B*.

### Detection of Y-chromosome probes

Seventeen public 450K datasets comprising 2826 samples representing a wide range of tissues were downloaded from the Gene Expression Omnibus (GEO) repository. Two thousand seven hundred fifty-five samples (described in Table 1) passed our comprehensive quality control pipeline including screening to exclude sex mismatches or failed assays according to any of the 17 performance metrics outlined in the Illumina BeadArray Controls Reporter Software Guide as implemented in the *ewastools* package [5]. Samples were grouped by sex (male/female = 1313/1442), and the number of detected Y-chromosome probes was counted for a range of *p* value cut-offs. A good cut-off should classify most Y-chromosome probes among females as undetected while retaining most of them among males.

**Table 1:** Overview of 450K Gene Expression Omnibus datasets used in the current study

GEO Accession	Tissue	Male (*n*)	Female (*n*)
GSE60655	Vastus lateralis muscle	16	20
GSE61496	Whole blood	154	141
GSE63106	Cartilage from knees and hip joints	24	38
GSE65163	Nasal epithelial cells	35	36
GSE69502	Fetal tissues: muscle, kidney, spinal cord, brain, chorionic villi	89	81
GSE74432	Whole blood	57	62
GSE75196	Placenta	11	13
GSE75248	Placenta	155	162
GSE85042	Cord blood	32	39
GSE85566	Airway epithelial cells	36	78
GSE86961	Papillary thyroid tumor tissue, non-neoplastic adjacent tissue	21	60
GSE87571	Whole blood	341	389
GSE89251	CD4+ T cells	38	98
GSE90871	Developing dorsolateral prefrontal cortex	13	11
GSE97362	Whole blood	145	83
GSE99863	Whole blood	126	117
GSE102177	Whole blood	20	14

### Technical replicates

A subset of 11 whole blood samples and their technical replicates (part of GEO Accession GSE61496 and GSE99863) were used to assess how detection *p* value cut-offs relate to precision and the prevalence of large technical outliers that can severely impact downstream analyses. The absolute difference in methylation levels between paired measurements was compared across detected and undetected probes after QC and preprocessing [6]. Large outliers were defined as measurements from a pair of technical replicates with an absolute difference larger than 20 percentage points, a substantial difference outside the confidence interval for the measurement precision of this technology [7].

### EWAS setting

Finally, we evaluated the impact on downstream analyses: a dataset of 732 peripheral blood samples with individuals aged 14 to 94 years from a population-based cohort (GEO Accession GSE87571, [8]) was used to conduct an EWAS with chronological age. Seven hundred twenty-nine samples passed QC. Raw fluorescence intensities were corrected for dye bias using the RELIC method [6], but no normalization was performed. Methylation levels of all autosomal CpG sites were regressed linearly on age, sex, leukocyte composition, and the assay performance metrics mentioned above. Methylation levels were expressed as *β* values because of their linear relation with cell proportions. Proportions of seven major leukocyte types were estimated using the algorithm developed by Houseman et al. [9] trained on the combination of two reference datasets of purified cell types [10, 11]. Information on the 96-well plate on which samples were allocated (and bisulfite converted) was not included as this important batch variable could not be reconstructed from GEO metadata alone.

Code to reproduce all analyses is available at doi.org/10.5281/zenodo.1443561, and a function to calculate detection *p* values based on non-specific fluorescence, including for *minfi* objects, can be found in the *ewastools* package (github.com/hhhh5/ewastools).

## Results

### Detection of Y-chromosome probes

**Fig. 1:**
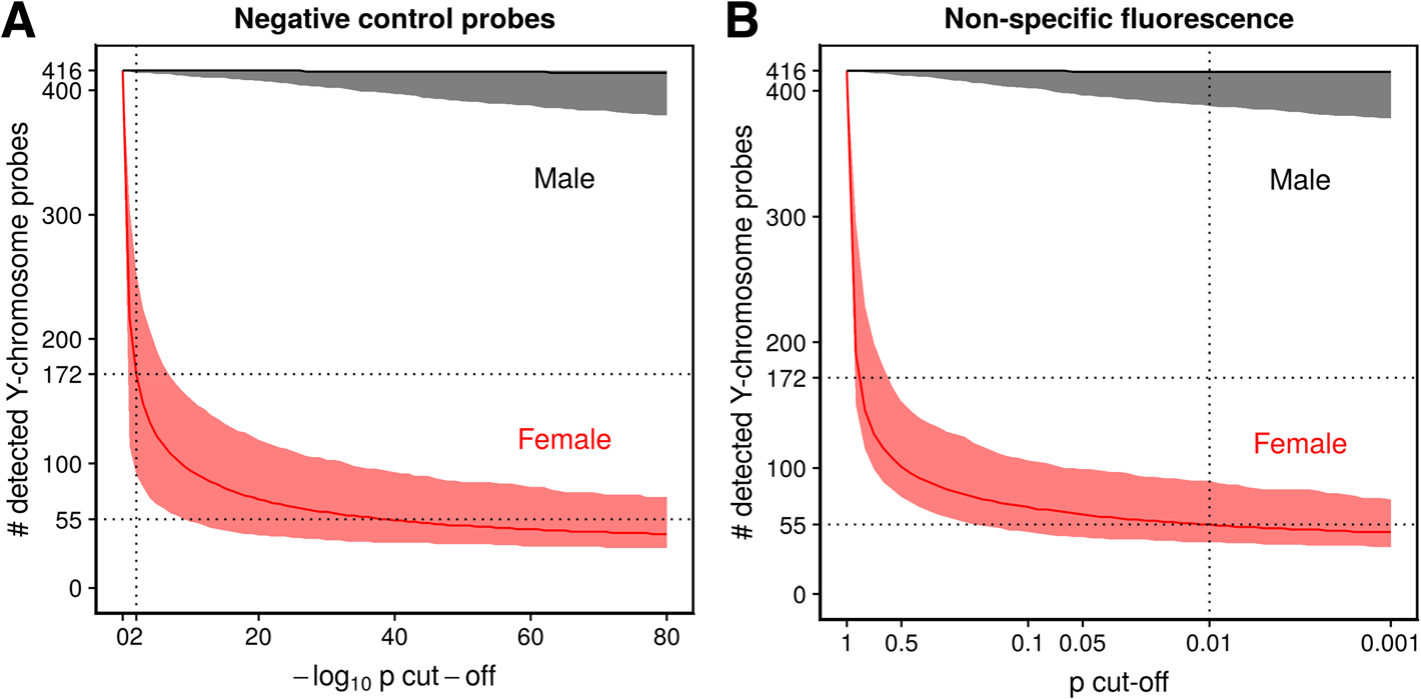
Choosing the right cut-off. Median number (with 2.5th and 97.5th percentiles) of detected Y-chromosome probes among 1313 male and 1442 female samples for a range of detection *p* value cut-offs. Negative control probes were used to estimate the background noise distribution in the left panel, whereas non-specific fluorescence was used in the right panel. The cut-off 0.01 (corresponding to 2 in the left panel due to the transformed *x*-axis) is highlighted

When estimating *B* using Illumina’s built-in negative control probes (NEG), we evaluated potential cut-off choices ranging from 1e00 to 1e−80, and for the higher non-specific fluorescence (NSP) intensities, we evaluated cut-off choices from 1e00 to 1e−03 as they resulted in higher detection *p* values. Among the 416 total Y-chromosome probes on the 450K array, the conventional and most widely used NEG/0.01 filter resulted in a median of 172 probes (41%) called detected among samples from females, whereas a NSP/0.01 filter resulted in a much lower median of 55 probes (13%) per sample. Among samples from males, the median number detected was 416 (100%) and 415 (100%) for NEG/0.01 and NSP/0.01, respectively. Figure 1 shows the median number (and 2.5th and 97.5th percentiles) of detected Y-chromosome probes for all evaluated cut-offs.

**Fig. 2:**
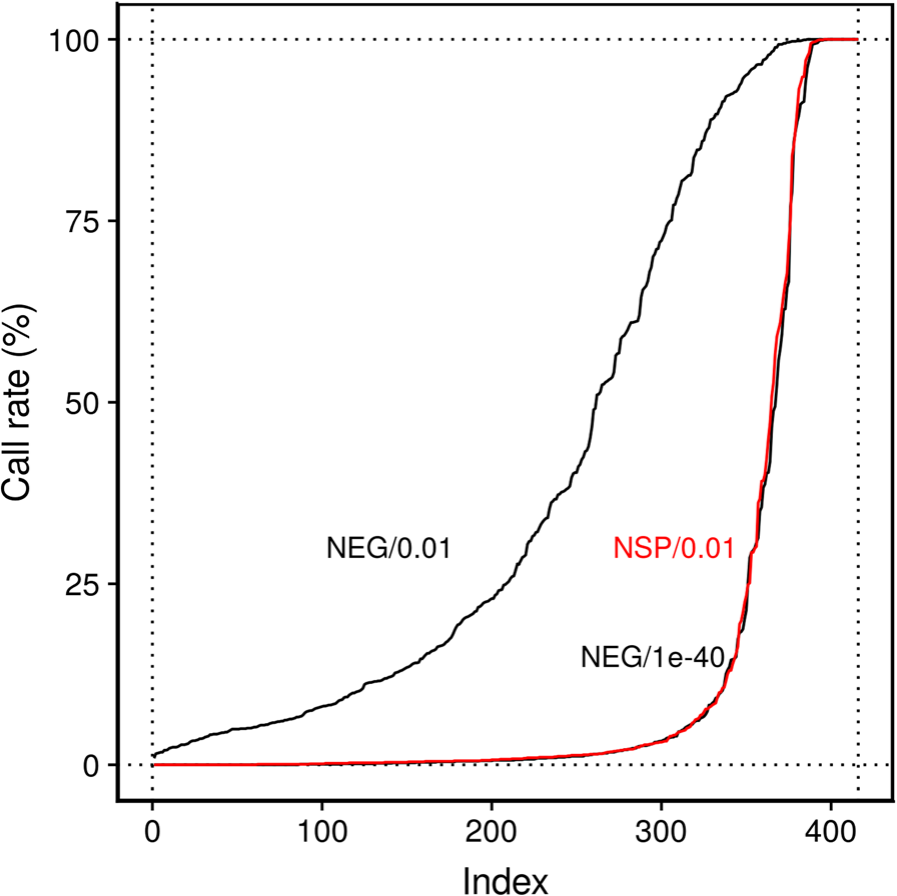
Detecting what is not supposed to be there. Call rates of Y-chromosome probes among the 1442 female samples for three approaches to classifying detected probes. Probes are ordered on the *x*-axis by increasing call rate (order not identical between curves). Only six probes had a call rate < 2% when using the conventional cut-off of 0.01 with the background distribution estimated from negative control probes (NEG/0.01). For more stringent criteria (NSP/0.1 and NEG/1e−40), there is an almost clear-cut separation between undetected and detected probes including some cross-hybridizing to autosomal CpG sites

Evaluating the proportion of times each Y-chromosome probe was detected (call rate) among all 1442 female samples revealed very distinctive patterns depending on the chosen filter (Fig. 2). When applying a more stringent filter (including NSP/0.01), there was a separation between those probes called almost always detected or undetected, respectively. The former category probably represents probes that are cross-reactive with autosomal loci, as 26 (87%) of the 30 probes with a call rate > 98% were listed by Chen et al. [12] as cross-reactive as determined by sequence homology, whereas only 38 (14%) of the 275 probes with a call rate < 2% were. In contrast, with the more permissive NEG/0.01 filter, only 6 of the Y-chromosome probes (none of them cross-reactive) had a call rate < 2% among females. To achieve results as stringent as the combination NSP/0.01 using negative control probes would require a cut-off around 1e−40, far below the previous recommendation and most stringent previously considered value of 1e−16 by Lehne et al. [1]. Using more stringent criteria also led to discarding a larger proportion of observations among the remaining probes (evaluated in all 2755 samples): NEG/0.01 resulted in a median of 84 undetected probes per sample among all autosomal and X-chromosome probes. Switching to NSP/0.01 resulted in a median of 674 undetected probes per sample, approximately 0.14% of the data.

### Technical replicates

Across 11 pairs of technical replicates, the median difference in methylation was 1.8 pp (percentage points) among the probes deemed detected in both index sample and replicate, and 9.5 pp (NEG/0.01) and 8.0 pp. (NSP/0.01) for all other pairs, respectively. Eleven thousand eight hundred seventy-seven probe pairs were large outliers with a difference > 20 pp, of which 743 (6%) (NEG/0.01) and 3602 (30%) (NSP/0.01) were classified as undetected in either sample, respectively.

### EWAS setting

**Fig. 3:**
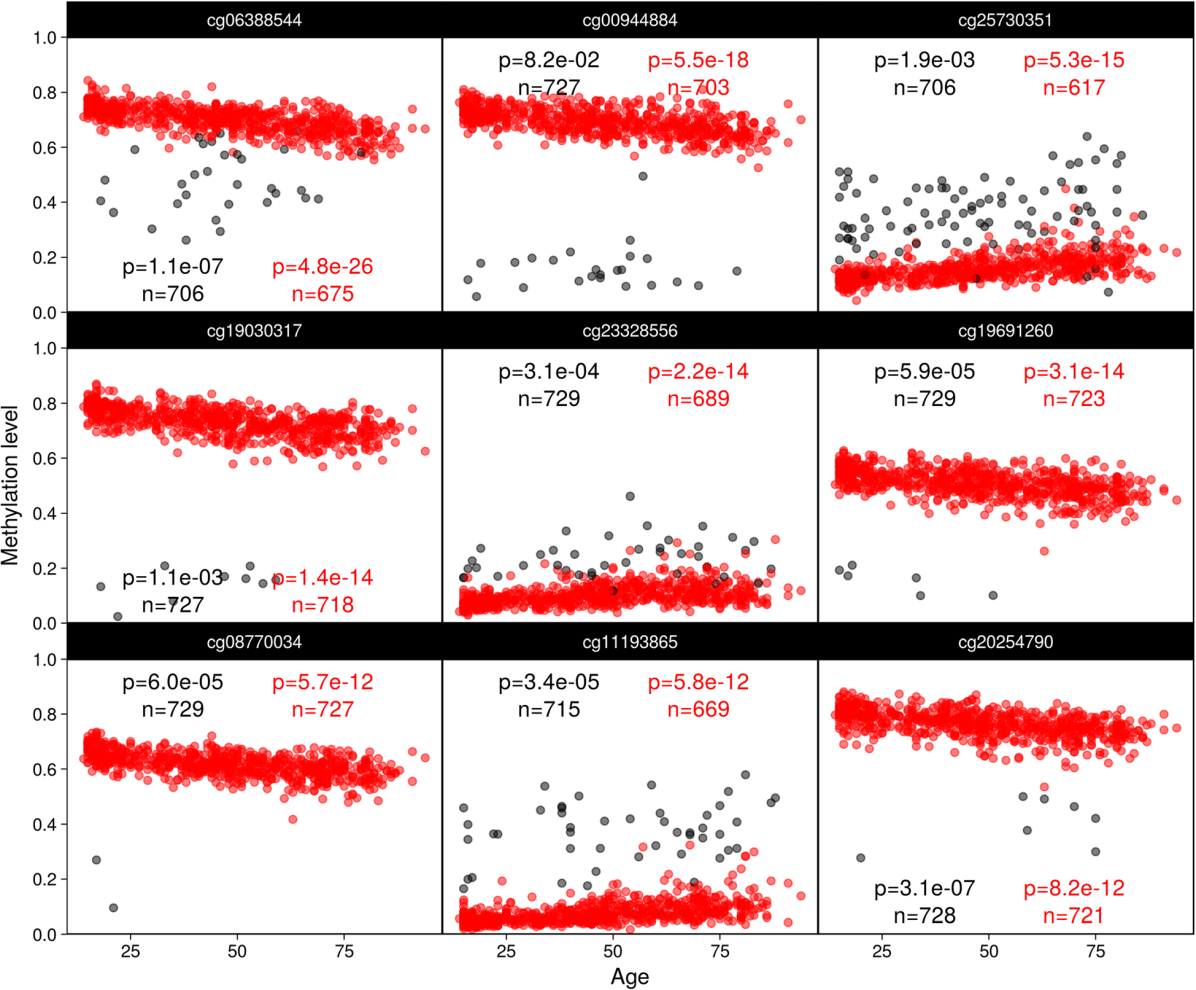
Previously obscured associations. Results from a reanalysis of a previously published epigenome-wide association study. Top nine uncovered associations between chronological age and DNA methylation levels (red) in peripheral blood reaching significance (relative to a Bonferroni threshold of 1.06e−07) after dropping observations (black) passing the more permissive NEG/0.01 cut-off but failing the more stringent NSP/0.01 cut-off. Annotations represent raw *p* values and sample sizes

**Fig. 4:**
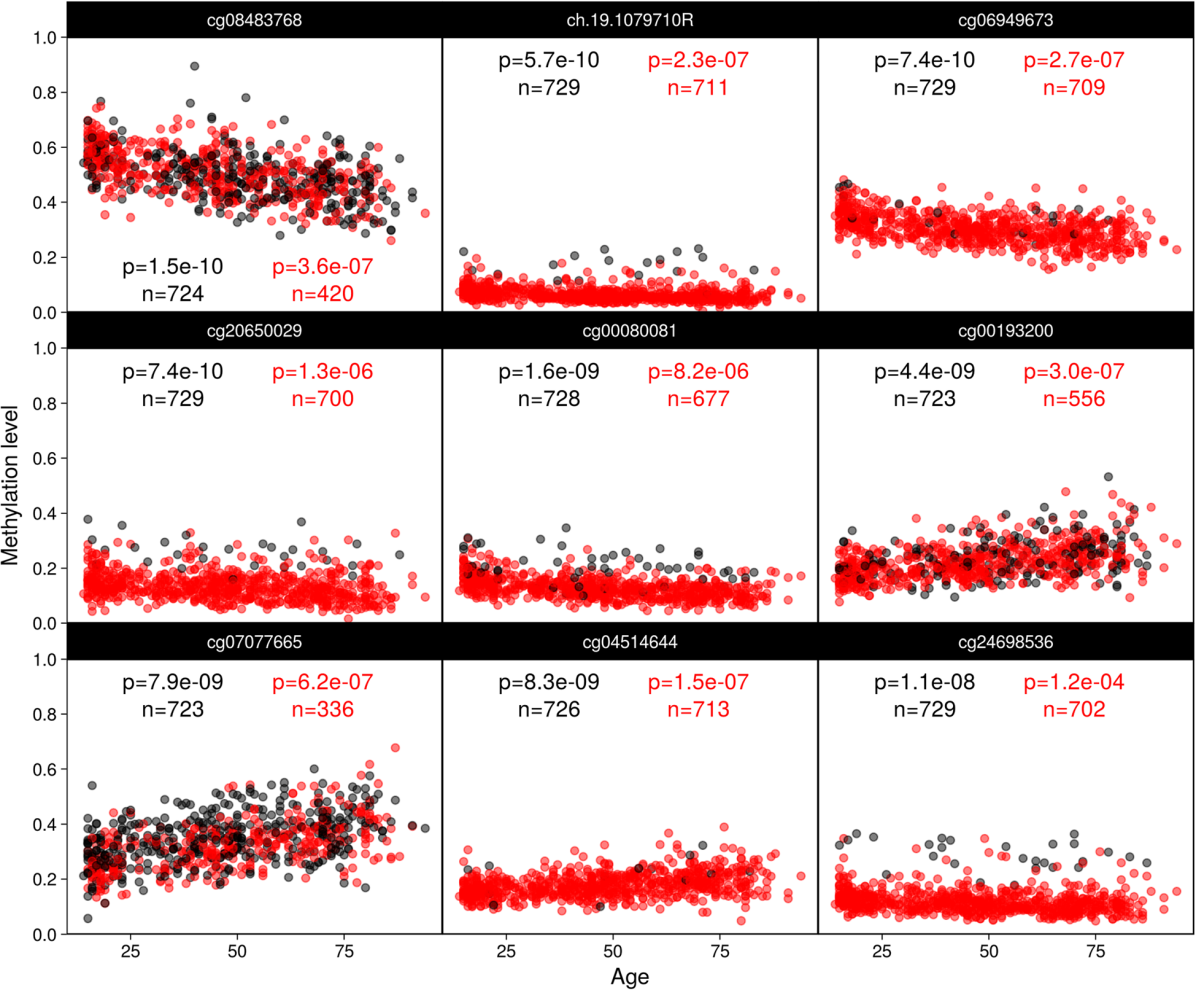
Associations losing significance. Results from a reanalysis of a previously published epigenome-wide association study. Top nine associations between chronological age and DNA methylation levels (red + black) in peripheral blood losing significance (relative to a Bonferroni threshold of 1.06e−07) after dropping observations (black) passing the more permissive NEG/0.01 cut-off but failing the more stringent NSP/0.01 cut-off. Annotations represent raw *p* values and sample sizes

Testing the association of 473,864 autosomal CpG sites with chronological age resulted in 38,799 hits for NEG/0.01 and 38,847 hits for NSP/0.01, significant at the 5% level, respectively, after Bonferroni adjustment of *p* values for multiple comparisons. Forty-four hits were found only for NEG/0.01 and 92 hits were found only for NSP/0.01 while 38,755 were common to both. Importantly, both approaches only differ in which observations are deemed undetected while *β* values of the retained observations are identical. Figure 3 shows the top nine out of the 92 hits reaching Bonferroni significance (uncorrected *p* values < 1.06e−07) only after applying NSP/0.01, demonstrating how even small numbers of spurious values included under current practices (NEG/0.01) contaminate the distribution of observed methylation *β* values and can obfuscate even strong associations, as in some instances the statistical significance jumps many orders of magnitude (e.g., from 1.1e−07 to 4.8e−26 for cg06388544 and from 8.2e−02 to 5.5e−18 for cg00944884). The top nine of the 44 former hits, i.e., associations losing significance (uncorrected *p* values < 1.06e−07 for NEG/0.01 but not NSP/0.01), are shown in Fig. 4. It is unclear how many constitute either false positives turned true negatives (which seems to apply to ch.191079710R and cg24698536) or true positives turned false negatives (as it might be the case for cg08483768 or cg00193200). What is apparent however is that the change in significance is not as stark as for the opposite events.

## Discussion

Background subtraction/correction is a common preprocessing step for 450K and EPIC data. Whereas some methods utilize negative control probes to estimate a background noise distribution, others employ so-called out-of-band intensities for this task [13]. Yet both show very distinct distributions: negative control probes are reportedly designed not to match the human genome (although their probe sequences are proprietary) and consequently feature very low intensities; in contrast, many probes possess some sequence similarity with off-target loci resulting in non-specific or off-target binding, with the out-of-band intensities being on the lower side of the spectrum (yet higher than the negative controls) and the set of cross-reactive probes being on the other. The argument for choosing out-of-band intensities over negative controls for background correction is that the former better reflect background noise, as non-specific binding represents unwanted signal as well. And indeed, while detection *p* values were so far based on a background noise distribution estimated from negative control probes, a recent paper proposes switching to out-of-band intensities [14]. Zhou et al. show that their approach increases precision and protects against false-positive findings, e.g., genetic deletions in tumor samples being mistaken for epigenetic silencing.

Similarly to Zhou et al., we propose swapping out negative control probes for another set of probes better suited to estimate the background noise distribution. However, we recommend against using out-of-band intensities as these, even though of Infinium Type I design, may equally suffer from cross talk [15] just like the probes of Infinium Type II design: e.g., in the case of a completely methylated CpG site targeted with a probe of Type I design and designated to be measured in the red color channel, the high concentration of red dye (Cy5) bound to the bead targeting the methylated variant may leak into the green—here the out-of-band—color channel, thereby inflating the observed intensity as we noted when comparing the out-of-band intensities of fully methylated and unmethylated probes. By restricting the set of probes used to estimate the background distribution to those for which no high concentration of the opposite fluorophore is to be expected, we avoid overestimating the background fluorescence distribution due to cross talk. In addition to other implementation differences with Zhou et al., our EWAS reanalysis demonstrates that a more stringent filtering by detection *p* values can not only protect against false-positive findings, but against false-negative findings as well.

We recommend to utilize non-specific fluorescence to estimate the background distribution and a cut-off of 0.01 as this combination (NSP/0.01) classified most Y-chromosome probes in female samples as undetected while still calling almost all Y-chromosome probes among males. The fact that the conventional and widely used filter NEG/0.01 led to so many calls of Y-chromosome probes among females demonstrates that negative control probes do not faithfully reflect background noise levels and that derived detection *p* values are therefore not accurate, which is why an extreme cut-off such as 1e−40 was necessary to achieve similarly stringent results as NSP/0.01. The NSP/0.01 filter also helped to exclude many more (30% vs. 6%) of the large outliers (> 20 pp) between technical replicates while dropping only a small fraction (0.14%) of the overall data. It has become common practice in the methylation microarray literature to drop probes and even entire samples if the number of measurements deemed undetected exceeds a certain threshold on the grounds that such probes/samples are unreliable [16]. Such extreme measures might no longer be necessary with our proposed method as it provides a more stringent, but especially also more accurate, assessment of detection.

Although our method improves the detection of large outliers among technical replicates, the majority of large outliers still remained: this may indicate that there are other unknown mechanisms that create such spurious values besides low fluorescence. Most probes deemed undetected showed deviations between replicates smaller than 20 pp although their median absolute difference was still much larger than among detected probes indicating that they should be removed as imprecise measures. In our EWAS reanalysis, strong associations were revealed in probes which had a substantial proportion of observations dropped after filtering. Furthermore, with the exception of a few exposures such as smoking, effect sizes of epigenomic associations in whole blood are often very small and removing these least reliable data points may strengthen statistical power. DNA methylation microarray studies have found and validated associations with BMI [17], diabetes [18], and age, with the corresponding biomarkers showing effect sizes of a few percentage points. Our reanalysis of a large EWAS of age shows that the choice of detection *p* value cut-off impacts statistical inference as unfiltered spurious values can obfuscate even strong associations with the discovery of weak associations presumably being even more impeded, supporting the need for more stringent data preprocessing. It should be noted that this conclusion holds regardless of whether the associations with chronological age discovered here are genuine or the result of residual confounding. However, the uncovering of new associations here comes with a tradeoff as a smaller number of sites lose significance. In some instances, this may include false positives previously driven by now discarded outliers, but other probes may decrease significance due to a reduced sample size. While our proposal represents an improvement and reasonable tradeoff over the current implementation of detection *p* values, there may be further room left to discriminate between reliable and unreliable observations.

## Conclusions

A more stringent preprocessing of microarray DNA methylation data is required to filter out spurious values. We demonstrate that restricting to measurements that pass our new detection *p* value function greatly decreases the prevalence of large outliers that can drive false-positive findings and can avoid false-negative findings. Our R package *ewastools* provides the necessary functions following our recommendations and is compatible with raw .idat files or *minfi* processing pipelines.

## Abbreviations

450K: Illumina Infinium HumanMethylation450 BeadChip
BMI: Body mass index
EPIC: Illumina Infinium MethylationEPIC BeadChip
EWAS: Epigenome-wide association study
GEO: Gene Expression Omnibus
NEG: Negative control probes
NSP: Non-specific fluorescence
QC: Quality control
